# Ultrasound-Assisted Eutectic Solvent-Based Process Intensification for Sustainable Recovery of Oleuropein from Olive Leaves

**DOI:** 10.3390/molecules30183829

**Published:** 2025-09-21

**Authors:** Andrea Sánchez-Monedero, María González-Miquel, Emilio J. González

**Affiliations:** Dpto. Ingeniería Química Industrial y del Medio Ambiente, ETSI Industriales, Universidad Politécnica de Madrid, C/José Gutiérrez Abascal 2, 28006 Madrid, Spain; andrea.smonedero@upm.es (A.S.-M.); maria.gonzalezmiquel@upm.es (M.G.-M.)

**Keywords:** olive leaves, solid/liquid extraction, green solvents, natural eutectic solvents, polyphenols, ultrasound-assisted extraction, oleuropein

## Abstract

Olive leaves, a significant source of agri-food waste, can be valorized as feedstock in biorefineries due to their high content of antioxidant compounds, mainly polyphenols. This study aims to valorize olive leaves through an efficient solid/liquid extraction of oleuropein, its main polyphenol, using green solvents and advanced technologies. Accordingly, three natural eutectic solvents formed with 1,2-butanediol and choline chloride, betaine, or proline, which incorporated water or ethanol as cosolvents, and ultrasound-assisted extraction to enhance the process were used in this work. Additionally, the effect of the cosolvent composition on the physical properties of the solvent mixtures (i.e., density and viscosity as a function of temperature) was evaluated. The extraction time was optimized for both conventional and intensified extractions, and the antioxidant activity of the extracts was assessed over time to determine their stability. Measurements through high-performance liquid chromatography and antioxidant activity assays concluded that ultrasound-assisted extraction using the solvent proline:1,2-butanediol prepared with ethanol at 25–75% composition yielded the best results at 37.00 mg of oleuropein per gram of dry sample (g/ds), obtained after just 1 min of intensified extraction, with a notable reduction in both time and energy consumption from conventional extraction, while providing significant antioxidant activity and stability.

## 1. Introduction

Polyphenols are a diverse and multifunctional group of bioactive substances reported in plants as secondary metabolites. They represent the second most abundant group of organic compounds (just after cellulose) in plant structure, taking different roles in them, such as structural support or the scavenging of reactive oxygen species (ROS) [1]. Their capacity to remove ROS of polyphenols, possibly due to the antioxidant activity of these compounds, plays an essential part in ultraviolet solar radiation protection and anti-allergenic, antiatherogenic, anti-inflammatory, antibacterial, antiviral, anti-diabetic, anti-thrombotic, chemopreventive, cardioprotective, and vasodilatory effects of plant-based treatments [2,3,4,5,6]. As a result, polyphenols and polyphenol extracts have become high-added-value components widely used in food, medicine, cosmetics, and pharmaceuticals, amongst other fields [7,8,9].

**Figure 1:**
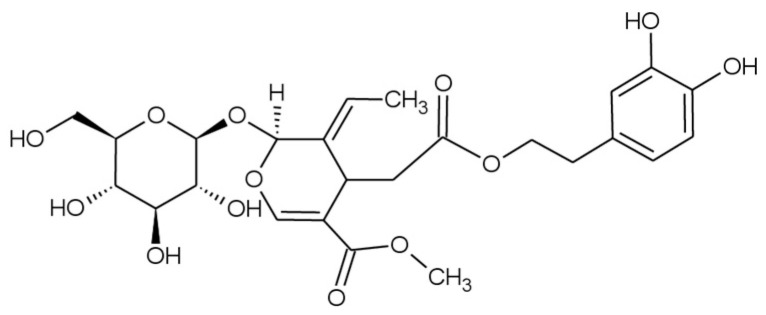
Structure of oleuropein.

Olive trees have a wide variety of polyphenols that not only appear in the olive fruit and its oil but are also found in other parts of the tree, including leaves and twigs [10]. The major polyphenol found in olive leaves is the oleuropein (Figure 1), a secoiridoid with multiple reported biological and pharmacological activities, including anticancer, cardioprotective, neuroprotective, gastroprotective, hepato-protective, anti-diabetes, anti-obesity, and radioprotective effects. These properties are largely attributed to its antioxidant and anti-inflammatory effects. Furthermore, the high antioxidant capacity of oleuropein may contribute to the treatment of several pathologies associated with oxidative stress, such as atherosclerosis, cancer, aging, rheumatoid arthritis, and inflammation [11]. According to recent estimates from the Food and Agriculture Organization Corporate Statistical Database (FAOSTAT), the world area dedicated to olive crop in 2023 was just above 11.1 Mha, with Spain being the leading producer of olives worldwide at more than 2.6 Mha cultivated (23.8% of the total global area dedicated to olive trees) [12]; hence, olive leaves constitute an extensive bio-agricultural waste from the olive industry that is usually burnt, thrown away, or ground and scattered on the field after the pruning process [13], potentially causing harmful effects in the environment, due to their high organic content and phytotoxicity, and increasing the cost for producers due to their removal, storage, and elimination [11,14]. Because of this, olive leaves represent an abundant but undervalued resource that could be valorized through the recovery of oleuropein.

Extraction is a critical stage in the isolation, identification, and use of polyphenols [15]. However, there are many aspects of this process to consider, from the choice of extraction solvent to the process parameters and the cost in terms of time and energy of the whole process [16,17]. Traditional extraction methods, i.e., maceration, heat treatment, Soxhlet extraction, enfleurage method, etc., are known to consume large amounts of solvent, energy, and time, leading to a high process cost. Hence, several alternative extraction methods, such as ultrasound-assisted extraction, microwave extraction, supercritical extraction, high-pressure extraction, and enzyme-assisted extraction, have been studied to replace them. These alternatives are considered favorable as they consume less energy, time, or solvent, or they improve the safety of the extraction process to achieve a still high-quality extract [17].

Ultrasound-assisted extraction is an approach not only deemed safer and more environmentally friendly than conventional methods, but also an efficient and economically viable process in the extraction of polyphenols. This method of extraction is based on the concept of cavitation. Cavitation effects result from low-frequency ultrasound waves created in the 20–100 kHz range, which produce high power levels [18,19]. These high-power ultrasounds can modify some physico-chemical properties, such as the expansion and compression cycles that the ultrasound wave generates when passing through a medium, creating bubbles in a liquid, which can grow and finally collapse. This mechanism produces high localized temperature and pressure that permits the rupture of cell walls and other intermolecular bonds, enhancing the extraction process by increasing the solvent penetration and mass transfer without heating. Thus, this method allows the extraction of thermolabile components that are degraded at lower temperatures [20,21,22]. The advantages of the use of ultrasound energy for extraction include reduced extraction time and temperature, more effective mixing, faster energy and mass transfer, selective extraction, reduced equipment size, faster start-up, and increased production [23]. Ultrasound-assisted extraction has been used for the extraction of several different polyphenols from spinach [24], black tea [25], grape seeds [15,26], and several dessert plants [27] and barks [28,29].

On the other hand, a green alternative to these conventional solvents is the use of eutectic solvents (ES). ES are mixtures of two or more components that are characterized by a decrease in the lattice energy of the system, which promotes a lower melting point than the values of the pure forms of their constituents: a hydrogen bond donor (HBD) and a hydrogen bond acceptor (HBA) composed of Lewis or Brønsted acids and bases. Traditionally, all eutectic mixtures have been referred to as “deep eutectic solvents”, or DES, regardless of their nature. However, according to Abranches et al., deep eutectic solvents are only a group within eutectic solvents that exhibit negative deviations from thermodynamic ideality in their liquid phase [30]. Eutectic solvents are considered green alternatives due to their tunable chemical properties, which result in solvents with low melting points, low volatility, nonflammability, low vapor pressure, and chemical and thermal stability, as well as high biodegradability, low toxicity, easy production, and low costs, thus offering a more sustainable solvent alternative [31,32,33]. When the components of this mixture are derived from natural sources, the liquid is named NAES, meaning natural eutectic solvent. These components are usually sugars, alcohols, sugar alcohols, organic acids or bases, amino acids or amines that occur naturally in the metabolism of living organisms. And so, the use of NAES entails further development of the environmental and toxicity risks compared with common eutectic solvents [34,35]. However, the high viscosities of most eutectic solvents can limit mass transfer, which results in a reduction in the extraction efficacy of the process. Frequently, to resolve this drawback, a low viscosity cosolvent, such as water, is added to the ES, reducing the overall viscosity of the system [36]. The use of natural eutectic solvents in the solid/liquid extraction process to obtain polyphenols from biomass has been widely established in the literature. In recent years, polyphenol extraction has been studied from apple pomace [37], grape seeds [38], sour cherry pomace [39], spent coffee grounds [40], grapevine cane [41], or different plant leaves [42,43,44,45].

The combination of the ultrasound-assisted extraction technique with the use of natural eutectic solvents presents a potential eco-friendly improvement of the conventional extraction process. There have been recent studies that explore the combination of these techniques for the extraction of bioactive compounds from olive leaves, but mainly about the effect of different HBAs and HBDs and their ratios [46,47,48,49].

This work focuses on the study of the extraction of oleuropein from olive leaves using different natural eutectic solvents combined with either water or ethanol as a cosolvent at several different compositions while also studying the effect of using ultrasound-assisted extraction compared to the use of the conventional one and the effect of the extraction time on the oleuropein extraction efficiency. Also, an additional objective of this work is the study of the degradation through time of the antioxidant activity of these extracts.

## 2. Results and Discussion

### 2.1. Solvent Characterization

As the first step, the natural eutectic solvents/cosolvent mixtures used in this study were characterized in terms of density (ρ) and dynamic viscosity (η). These mixtures were prepared by combining a NAES, formed by mixing choline chloride (ChCl), betaine (Bet), or proline (Pro) with 1,2-butanediol (2B) in a molar ratio of 1:4, with water or ethanol at different volume ratios, as shown in Table 1.

**Figure 2:**
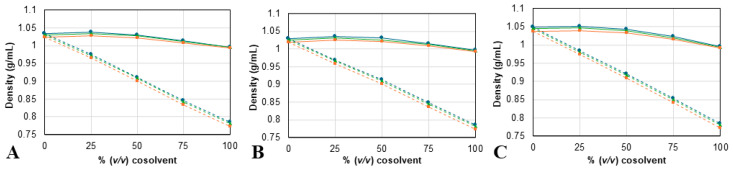
Density measurements for mixtures of (**A**) ChCl:2B, (**B**) Bet:2B, and (**C**) Pro:2B, with water (continuous line) or with ethanol (dotted line) obtained at three different temperatures: 25 (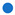), 30 (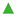), and 40 °C (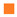). ChCl: chlorine chloride, Pro: proline, Bet: betaine, 2B: 1,2-butanediol.

**Figure 3:**
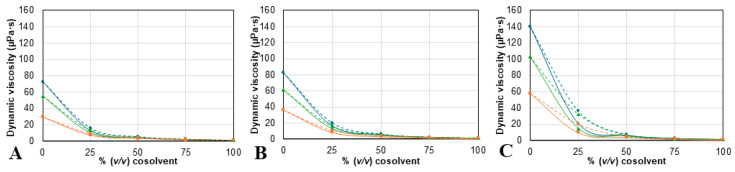
Dynamic viscosity measurements for mixtures of (**A**) ChCl:2B, (**B**) Bet:2B, and (**C**) Pro:2B, with water (continuous line) or with ethanol (dotted line) obtained at three different temperatures: 25 (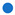), 30 (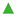), and 40 °C (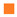). ChCl: chlorine chloride, Pro: proline, Bet: betaine, 2B: 1,2-butanediol.

The density (ρ) and dynamic viscosity (η) of each NAES/cosolvent mixture were measured at different temperatures (from 25 to 40 °C), to physically characterize the different solvents used in this study, since both of these properties greatly impact the extraction efficiency. The experimental values of both properties are summarized in Table S1, available as Supporting Information, and shown in Figure 2 and Figure 3.

The results show a dependence on temperature and composition from both ρ and η values. For all mixtures, density and viscosity values decrease as the temperature increases. Also, ρ values (Figure 2) change linearly as more ethanol is added but have a maximum at 25% of added water. For the η values (Figure 3), the mixtures containing ethanol exhibit a higher viscosity than the corresponding mixture with water, as was expected, since the viscosity of ethanol is higher than the viscosity of water. Furthermore, the results show that the solvent prepared with proline has higher ρ and η values; meanwhile, the solvents containing betaine have the lowest densities, and choline chloride ones have the lowest viscosities of the three. Despite this, the differences between solvents decrease as the cosolvent content increases. The results for both density and viscosity of the NAES/water and NADES/ethanol mixtures are consistent with previously reported tendencies [50,51,52].

### 2.2. Extraction Process Optimization

All the different mixtures of NAES with water or ethanol as cosolvents, the three pure NAES (ChCl:2B, Bet:2B, and Pro:2B), as well as pure water and ethanol, described in Table 1, were studied to determine their capacity to extract oleuropein, the main polyphenol present in olive leaves. All solvents were applied for conventional extraction (CE), and then an ultrasonic probe (ultrasound-assisted extraction, USAE) was used to intensify the extraction process.

**Figure 4:**
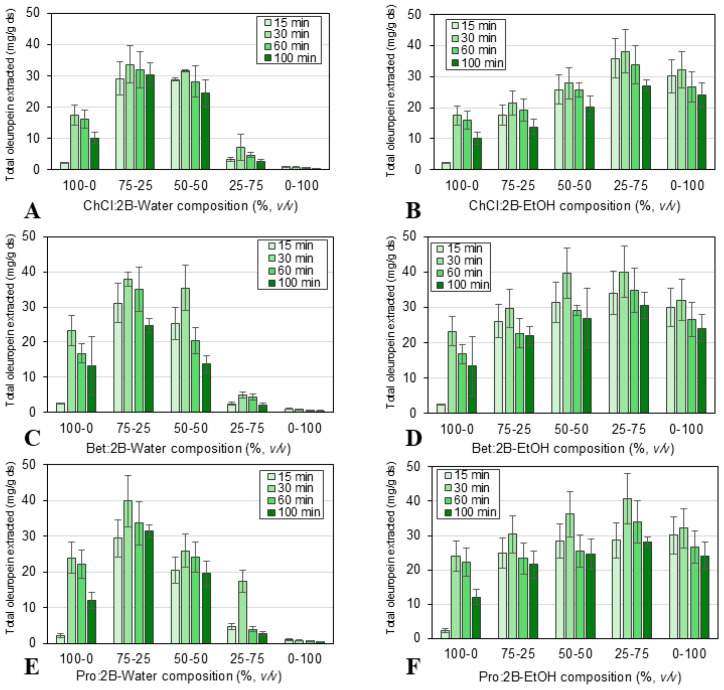
Effect of extraction time (15, 30, 60, 100 min) and solvent composition: (**A**) ChCl:2B and water mixtures, (**B**) ChCl:2B and ethanol mixtures, (**C**) Bet:2B and water mixtures, (**D**) Bet:2B and ethanol mixtures, (**E**) Pro:2B and water mixtures, (**F**) Pro:2B and ethanol mixtures; for the conventional extraction of oleuropein (mg/g ds) from olive leaves at 900 rpm, solid/liquid ratio 1:10 and 60 °C. Mean values ± standard deviation (mg oleuropein/g ds). ChCl: chlorine chloride, Pro: proline, Bet: betaine, 2B: 1,2-butanediol.

**Figure 5:**
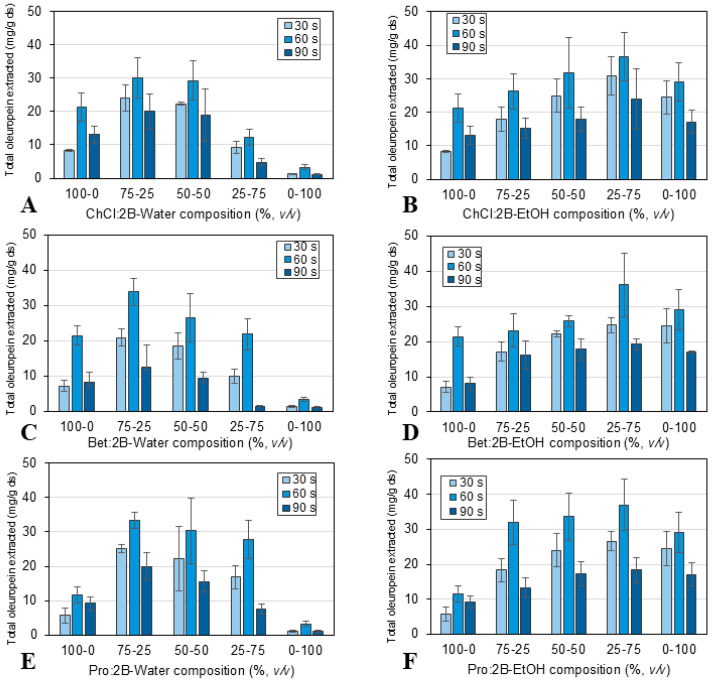
Effect of extraction time (30, 60, and 90 s) and solvent composition: (**A**) ChCl:2B and water mixtures, (**B**) ChCl:2B and ethanol mixtures, (**C**) Bet:2B and water mixtures, (**D**) Bet:2B and ethanol mixtures, (**E**) Pro:2B and water mixtures, (**F**) Pro:2B and ethanol mixtures; for the ultrasound-assisted extraction of oleuropein (mg/g ds) from olive leaves at 10 W, solid/liquid ratio 1:10 and room temperature. Mean values ± standard deviation (mg oleuropein/g ds). ChCl: chlorine chloride, Pro: proline, Bet: betaine, 2B: 1,2-butanediol.

For these studies, the olive leaf powder samples were combined with each solvent–cosolvent mixture as described in Section 3.3. Then, in each case, the extraction time, a rather relevant parameter for its contribution to the overall process time and cost, was studied. The effects of extraction time and solvent composition on the oleuropein extraction capacity are shown in Figure 4 for the conventional extraction and in Figure 5 for the ultrasound-assisted extraction. More detailed results are provided in Tables S2 and S3, respectively (see Supplementary Information).

Firstly, the total amount of oleuropein extracted using conventional extraction (at 15, 30, 60, or 100 min of extraction time, 60 °C, 900 rpm, and solid/liquid ratio 1:10) was measured by HPLC. The results, pictured in Figure 4, show that the extraction time significantly affects the extraction efficiency, obtaining the most favorable oleuropein yield at 30 min. While the lower extraction values at the lowest extraction time (15 min) indicate that the solid/liquid systems have not yet reached equilibrium, the lower extraction values at longer extraction times could be a result of the oxidation of the oleuropein because of air or light [53]. Therefore, a longer extraction time did not mean a higher extraction efficiency. The highest value of oleuropein extracted at the optimal extraction time (i.e., 30 min) amounted to 40.62 mg/g ds over all solvents studied. It was observed that the composition of the solvents also significantly affects the extraction efficiency, reaching similar maximum values for all three NAES-based solvents at the following compositions along the cosolvents: 75% NAES-25% water and 25% NAES-75% ethanol. After 30 min of extraction, the maximum values achieved were 39.83 mg oleuropein/g ds for 75% Pro:2B-25% water and 40.62 mg oleuropein/g ds for 25% Pro:2B-75% ethanol. While using ethanol as a cosolvent for eutectic mixtures has barely been explored in literature, the optimal composition obtained by mixing NAES and water (25% of water) was in good agreement with the results previously reported. In these studies, several different eutectic solvents were applied to the solid/liquid extraction of polyphenols, flavonoids, and other bioactive compounds, achieving the best results with 20–30% (*v/v*) of added water [54,55,56,57,58].

The possibility of enhancing the extraction of oleuropein from olive leaves using natural eutectic solvents was further explored using ultrasound as an assisted technology for process intensification. The extraction solvents were again the three NAES previously studied, combined in different proportions with water and ethanol. The extraction process was carried out using an ultrasonic probe or processor under the conditions described in Section 3.3: extraction times of 30, 60, and 90 s, room temperature, 10 W power, and a 1:10 solid/liquid ratio. Figure 5 shows the results obtained in terms of total oleuropein extracted per gram of dried sample. A similar trend to that of the conventional extraction was observed in the ultrasound-assisted extraction regarding the optimal mixture composition of the solvents, obtaining an optimal oleuropein extraction at 75% NAES and 25% water or 25% NAES and 75% ethanol, and with Pro:2B as the best of the three NAES. On the other hand, as a result of using the ultrasonic probe, it was possible to reduce the extraction time to an optimal value of 60 s, achieving similar results to the conventional extraction performed at 30 min. In particular, extraction yields of 33.83 mg oleuropein/g ds for 75% Pro:2B-25% water and 37.00 mg oleuropein/g ds for 25% Pro:2B-75% ethanol were achieved using ultrasound-assisted extraction for 60 s.

**Figure 6:**
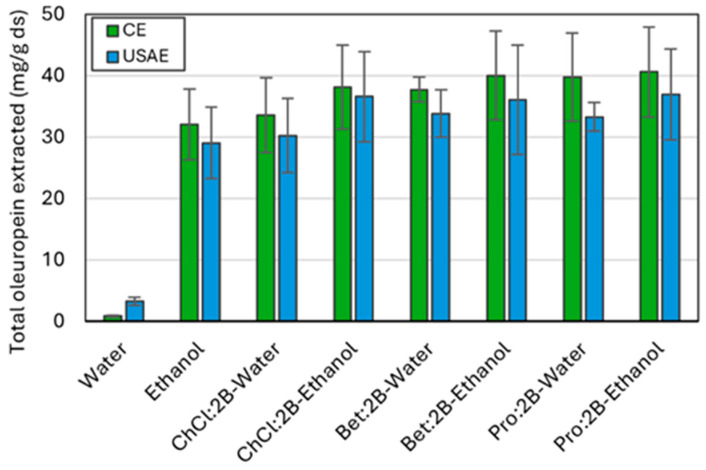
Total oleuropein extracted from olive leaves using conventional extraction (CE) and ultrasound-assisted extraction (USAE) for all three NAES-based solvents, water, and ethanol at the optimal conditions of extraction time (30 min at 60 °C and 900 rpm for CE and 1 min at room temperature and 10 W for USAE) and composition (25% of added water or 75% of added ethanol). Mean values ± standard deviation as mg oleuropein/g ds. ChCl: chlorine chloride, Pro: proline, Bet: betaine, 2B: 1,2-butanediol.

Figure 6 compares the optimal extraction conditions of the three NAES used in this work, with water and ethanol as references, both for the conventional method and the intensified one through ultrasounds. Optimal NAES–water and NAES–ethanol mixtures exhibit superior extraction efficiencies compared to pure water or ethanol, in both conventional and intensified extraction processes. This may be attributed to the decrease in viscosity of the NAES-based solvents when a cosolvent is added. Also, the extraction capacity of the ultrasonic method is slightly lower than that of the conventional method for all solvents (except water); however, the energy required when using the ultrasonic probe compared to the orbital shaker is significantly lower. Specifically, the extraction procedure using the ultrasonic probe for 60 s with an operating power of 10 W (which consumes about 33 h and 1200 kJ per kilogram of sample) results in a time reduction of up to 1.5 times and an energy reduction of up to 77 times compared to the extraction procedure using the orbital shaker for 30 min with an operating power of 515 W and a capacity for 20 samples (at about 50 h and 92,700 kJ per kilogram of sample). Therefore, using the ultrasound-assisted extraction method can be considered a substantial improvement in the extraction process.

### 2.3. Antioxidant Activity Degradation Study

Additionally, the evolution of the antioxidant activity of the extracts with time was also determined by performing the DPPH assay as described in Section 3.6 for 31 days. All six oleuropein-rich extracts obtained with the optimal mixtures of NAES–water and NAES–ethanol selected, as well as with pure water and ethanol as references, and through ultrasound-assisted extractions, were subjected to the analysis. After carrying out the solid/liquid extraction (1 min at room temperature using the ultrasonic probe, and a solid/liquid ratio of 1:10) and centrifugation with the eight solvents chosen, the extracts resulted were kept at room temperature and without sunlight, measuring their antioxidant activity at 0, 3, 7, 14, 21, and 31 days of storage time.

**Figure 7:**
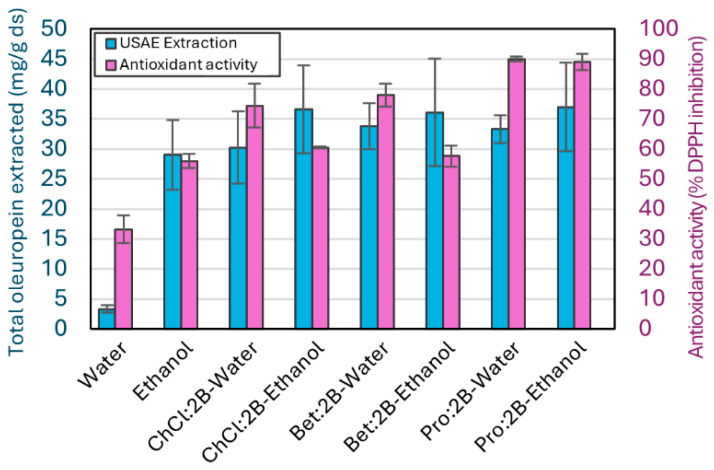
Extraction yields as total oleuropein extracted and as antioxidant activity in the per-centage of DPPH inhibition for all NAES–water and NAES–ethanol optimal mixtures obtained using the intensified method (USAE, 1 min at room temperature and 10 W), with water and ethanol as references. Mean values ± standard deviation as mg oleuropein/g ds for the extraction, and % DPPH Inhibition for the antioxidant activity. ChCl: chlorine chloride, Pro: proline, Bet: betaine, 2B: 1,2-butanediol.

Figure 7 presents both the oleuropein extracted and the antioxidant activity of each extract. Table S4, available as Supporting Information, includes all the experimental data obtained from the DPPH assays. Even though the extraction capacity is very similar for all six NAES–cosolvent mixtures, the DPPH assay shows a higher antioxidant activity for extracts using the Pro:2B NAES, with both water (90.0 ± 0.8%) and ethanol (89.0 ± 2.7%). This could be due to the Pro:2B solvent extracting other compounds from the olive leaves that are in a minority composition but help to enhance the antioxidant activity. On the other hand, mixtures with water result in a higher antioxidant activity of the extracts than the mixtures with ethanol, which is the opposite of what the extraction results show. Similarly to the results of Pro:2B solvents, the higher values of antioxidant activity for solvents that contain water instead of ethanol might be due to the lower selectivity of water during the extraction process, resulting in a higher concentration of minority compounds with high antioxidant activity. Also, since ChCl:2B and Bet:2B NAES have a significantly lower antioxidant activity than Pro:2B, the effect of the cosolvent is more evident in their extracts. Specifically, ChCl:2B–ethanol and Bet:2B–ethanol mixtures result in a very similar antioxidant activity to pure ethanol (60.5 ± 0.4%, 57.7 ± 3.5%, 56.0 ± 2.3%, respectively), which is consistent with their composition of 75% *v/v* of ethanol. On the other hand, the mixture of these NAES with water results in a higher difference between the mixtures and the pure water (74.5 ± 7.4%, 78.0 ± 3.8%, 33.2 ± 4.6%, respectively), a difference similar to that of the oleuropein extraction results.

**Figure 8:**
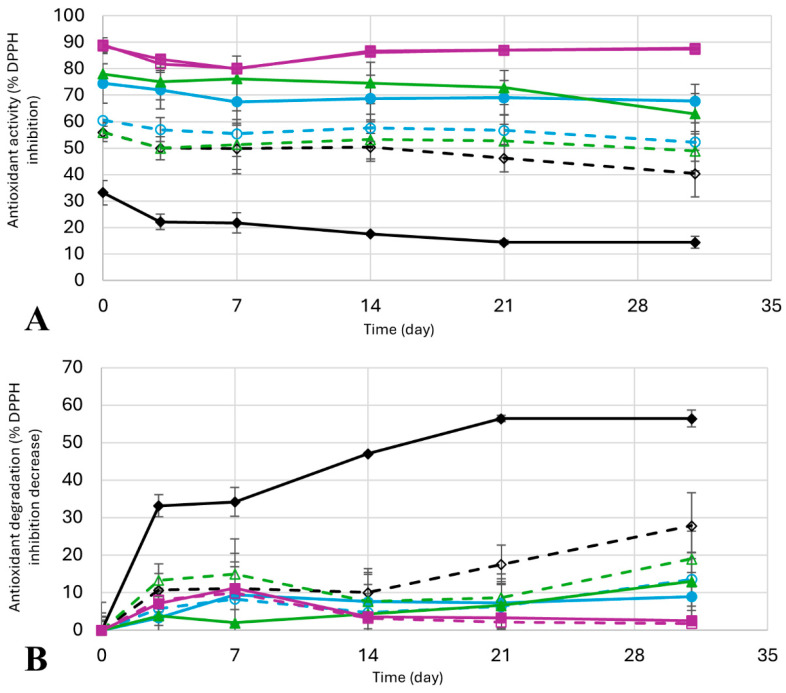
Degradation of antioxidant activity: Mean values ± standard deviation as (**A**) Percentage of antioxidant inhibition using DPPH, and (**B**) percentage of antioxidant activity degradation, as relative values of the DPPH inhibition decrease. Results for water (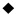, solid black line), ethanol (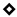, dotted black line), ChCl:2B–water (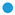, solid blue line), ChCl:2B–ethanol (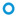, dotted blue line), Bet:2B–water (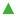, solid green line), Bet:2B–ethanol (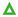, dotted green line), Pro:2B–water (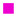, solid pink line), and Pro:2B–ethanol (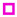, dotted pink line). ChCl: chlorine chloride, Pro: proline, Bet: betaine, 2B: 1,2-butanediol.

Finally, the stability of the antioxidant activity in all 8 extracts mentioned was studied through time (Figure 8, Table S4). The difference in antioxidant activity after 31 days in storage in absolute values (Figure 8A) is not very significant, with water being the solvent that loses more antioxidants (from 33.2 ± 4.6% to 14.5± 2.3%, almost a 20% in antioxidant activity of difference) and Pro:2B–water and Pro:2B–ethanol with almost no difference in antioxidant activity after 31 days (90.0 ± 0.8% to 87.7 ± 0.0%, and 89.0 ± 2.7% to 87.4 ± 0.3%). Taking into account the initial values of antioxidant activity of each extract, the difference in total degradation of antioxidant activity, or, in other words, the relative value of the antioxidant degradation (Figure 8B), shows that extracts obtained with water lose more than half their antioxidant activity after a month in storage (56.4 ± 7.0%). In comparison, Pro:2B–water and Pro:2B–ethanol only lose about 3% and 2%, respectively. Regarding the rest of the extracts, the degradation outcome is intermediate, as they lose from 7% to 16% of the antioxidant activity in absolute values, which translates to 9% to 28% of the degradation percentage. These results are consistent with the literature, where the use of natural eutectic solvents has been shown to improve the stability of phlorotannins [59], anthocyanins [60], or other polyphenol extracts [61].

Therefore, considering physical properties such as density and viscosity, and chemical properties like composition, oleuropein extraction efficiency, the antioxidant activity of the extract, and its stability, for all NAES mixtures studied, Pro:2B–ethanol 25–75% was chosen as the best solvent to extract oleuropein from olive leaves through an ultrasound-assisted extraction at room temperature for 60 s and a 1:10 S:L ratio. In future studies, key aspects such as solvent recyclability, hazard assessment of the extracts, more comprehensive evaluation using green chemistry metrics, and potential applications of the extracts should be investigated to better assess the overall viability and sustainability of the process.

## 3. Materials and Methods

### 3.1. Materials

The plant material matrix used was constituted by olive leaves obtained directly from an olive tree in Getafe, Madrid (Spain). They were separated from the branches, washed with deionized water, and air-dried for 24 h. Then, the biomass was completely dried in an oven at 40 °C for another 24 h. After, the leaves were put in a grinder (Bosch coffee grinder TSM6A013B, BSH Hausgeräte GmbH, München, Germany) until a fine powder was obtained.

For the preparation of the natural eutectic solvents, the following compounds were used: choline chloride (99.0%, Sigma-Aldrich, Saint Louis, MA, USA. CAS No. 67-48-1), L-proline (99.0%, Sigma-Aldrich, CAS No. 147-85-3), betaine (98.0%, Sigma-Aldrich, CAS No. 107-43-7), and 1,2-butanediol (98.0%, Sigma-Aldrich, CAS No. 584-03-2). Additionally, deionized water (produced by a Lab-Ion-L2, water purification unit, Herka Lab-In, Kreuzwertheim, Germany) and ethanol (99.9%, Scharlab, Barcelona, Spain, CAS No. 64-17-5) were used as cosolvents. Oleuropein standard (98.0%, HPLC grade, Sigma-Aldrich, CAS No. 32619-42-4), acetonitrile (HPLC grade, Honeywell, Madrid, Spain, CAS No. 75-05-8), water (HPLC grade, Honeywell, CAS No. 7732-18-5), methanol (HPLC grade, Honeywell, CAS No. 67-56-1) and 2,2-diphenyl-1-picrylhydrazyl (DPPH) (Sigma-Aldrich, CAS No. 1898-66-4) were used in the analysis of extracts.

### 3.2. Solvent Preparation and Characterization

The natural eutectic solvents used in this study were chosen because of their high efficiency in the extraction of oleuropein from olive leaves found in a previous study [62]. They were formed by mixing choline chloride (ChCl), betaine (Bet), or proline (Pro) as HBA, with 1,2-butanediol (2B) as HBD, in a molar ratio of 1:4 (HBA: HBD). The mixtures were prepared by weighing the components using an analytical high-precision balance, Sartorius M-Power AZ124 (Göttingen, Germany), with a precision of ± 0.001 g. Both components were then mixed and heated to 60–80 °C while stirring at 350 rpm until a homogeneous and transparent liquid was formed (after about 30 to 60 min of stirring). Then, each solvent was mixed with a specific volume of deionized water or ethanol, which were used as cosolvents for the NAES. The composition of each solvent is shown in Table 1.

Regarding solvent characterization, the density (ρ) of all solvents was measured at 25, 30, and 40 °C using a digital vibrating-tube densimeter (Anton Paar DMA 500, Graz, Austria) with an experimental uncertainty of ±0.0003 g/cm^3^. This equipment has an internal temperature control with an accuracy of ± 0.1 K. Dynamic viscosities (η) were obtained using Equation (1).

η = ν·ρ(1)

from kinematic viscosities (ν) and densities (ρ) of each solvent. Kinematic viscosities were experimentally determined using several micro-Ubblelohde viscosimeters (Comecta, Barcelona Spain) with a measurement range from 0.4 to 800 cSt and a range of uncertainty from ± 0.35% to ± 0.50%. The viscosimeter was placed in a bath Selecta “VB-1423” (Barcelona, Spain) with temperature control to obtain viscosity measurements at 25, 30, and 40 °C.

### 3.3. Solid/Liquid Extraction of Oleuropein from Olive Leaves

For the solid/liquid conventional extraction (CE), 0.5 g of olive leaves powder and 5 mL of solvent were added into a 15 mL test tube. Then, the tubes were introduced into a shaking incubator Labnet VorTemp 1550 (Madrid, Spain) to perform the extraction process at 900 rpm, 60 °C, and different extraction times (15, 30, 60, 100 min), followed by 10-min centrifugation (Unicen 21 Centrifuge, Ortoalresa, Madrid, Spain) at 2860× *g* (4200 rpm). Then, to further improve the recovery process of antioxidants, the use of natural eutectic solvents mixed with water or ethanol was combined with ultrasound-assisted extraction (USAE), using an ultrasonic processor UP200Ht from Hielscher (Hielscher Ultrasonics GmbH, Teltow, Germany) of 26 kHz, at 10 W, room temperature (RT), solid/liquid ratio of 1:10, and three extraction times: 30, 60, and 90 s. All established conditions were based on previous articles published in this research group [63]. Every extraction assay (CE and USAE) was performed in duplicate, and aliquots of the liquid extracts were analyzed using high-performance liquid chromatography (HPLC) to quantify the target compounds.

### 3.4. Chromatographic Quantification

Chromatographic analysis of the oleuropein extracted from olive leaves via NAES/cosolvent mixtures were performed on the JASCO 4000 Series HPLC system (JASCO, Madrid, Spain) equipped with a photo diode array (DAD) detector. Samples were always diluted 1:10 in a 50/50 mixture of methanol/water and filtered through 0.45 μm nylon filters (Mervilab, Madrid, Spain) prior the analysis. The injection sample volume was 20 μL. The column used was a C18 (Fortis (Neston, UK), 250 × 4.6 mm, 5 μm) at room temperature (25 °C). The mobile phase consisted of a 1.25% (*v/v*) aqueous solution of acetic acid (eluent A) and acetonitrile (eluent B), under the following gradient elution sequence, at a 0.5 mL/min flow rate: 15% of eluent B initially; linear increase to 20% of B for 7 min, linear increase from 20% to 45% of B from 22 to 27 min, linear increase from 45% to 48% of B from 27 to 30 min, and finally linear decrease from 48% to 15% of B from 38 to 41 min. Total analysis per sample was performed in 48 min. Detection and identification of oleuropein was performed using a DAD detector at 270 nm. A HPLC chromatogram of the results is presented in Supplementary Figure S1 (available as Supporting Information).

Quantifying the extraction of the target compounds was carried out from the integration of the peak area and using the calibration curve prepared with standard solutions for this compound (Figure S2, available as Supporting Information). The results, expressed as mg of target compound per g of dried sample (ds), were obtained from Equation (2):

Oleuropein (mg/g ds) = [OLE]/m·V·10(2)

where [OLE] is the concentration of the phenolic compound (in mg oleuropein/mL), measured by HPLC, and calculated by the calibration curve; V of the solvent is the amount of solvent used during the extraction, expressed in L; m is the amount of dried sample used expressed in g; and 10 is the dilution factor. In all cases, HPLC analyses were performed in duplicate, and the results were expressed as mg oleuropein/g ds ± standard deviation.

### 3.5. Statistical Analysis

Results of oleuropein extraction from both the conventional and the ultrasound-assisted extractions were always expressed as mean ± standard deviation. To determine statistical significance (*p* < 0.05) between the different values studied within each factor affecting the process, multifactor ANOVA post hoc Tukey’s test was used. Each analysis parameter was determined by two experimental measurements. All statistical analyses were performed using STATGRAPHICS (v.19) software.

### 3.6. Antioxidant Stability of the Extracts

The extracts obtained at the optimal operating conditions and the better-performing mixtures of ChCl:2B–water, ChCl:2B–ethanol, Bet:2B–water, Bet:2B–ethanol, Pro:2B–water, and Pro:2B–ethanol, along pure water and ethanol as references, were stored at room temperature and kept away from light for 31 days. Variations in the antioxidant activity were measured using the DPPH assay free radical method [64]. The following experimental procedure was carried out: 100 μL of a diluted solution of the sample (1:10 in methanol) was mixed with 2.9 mL of DPPH radical methanolic solution (6 × 10^−5^ mol/L). After a 30-min incubation in the dark at room temperature, the absorbance at 515 nm was measured in a JASCO V-730 UV–Vis spectrophotometer (JASCO, Madrid, Spain). All measurements were performed in duplicate. The inhibition of the initial concentration of DPPH· radical was calculated, in percentage, according to Equation (3):

DPPH Inhibition (%) = (A_0_ − A_1_)/A_0_·100 (3)

where A_0_ represents the absorbance of methanol blank sample and DPPH reagent and A_1_ represents the absorbance of the extractant solvent sample and DPPH reagent.

## 4. Conclusions

Three natural eutectic solvents, formed with 1,2-butanediol and either choline chloride, betaine, or proline, were used in this work to extract oleuropein—a polyphenol with significant antioxidant properties—from olive leaves. Water and ethanol were used as cosolvents. Two extraction methods were applied: a conventional method using an orbital shaker and elevated temperature, and ultrasound-assisted extraction using an ultrasonic probe at room temperature.

The extraction process was optimized in terms of extraction time and solvent–cosolvent composition for both methods, with the best results obtained using the solvent containing proline:1,2-butanediol [1:4] NAES and ethanol at 25–75%, respectively. Under these conditions, 40.62 mg of oleuropein per gram of dry sample (g ds) was extracted after 30 min of conventional extraction, and 37.00 mg/g ds was obtained after just 1 min of intensified extraction using the ultrasonic probe. Considering the time reduction (up to 1.5 times) and the energy reduction (up to 77 times) of the intensified process to obtain a similar oleuropein extraction efficiency rate to the conventional one, this method was shown to improve the oleuropein recovery process significantly.

Moreover, the antioxidant activity and stability over time for each of the oleuropein-rich extracts obtained using this intensified method were measured by a DPPH inhibition assay for 31 days. This confirmed the choice of proline:1,2-butanediol [1:4] as the best NAES among those studied since the antioxidant capacity of the extracts obtained with these solvents remained stable over time.

Additionally, these results were complemented with an assessment of the physical characteristics of the solvent–cosolvent mixtures, measuring their density and viscosity at different temperatures.

## Figures and Tables

**Table 1:** Composition of each solvent used in this work. HBA: Hydrogen bond acceptor, HBD: Hydrogen bond donor, ChCl: chlorine chloride, Pro: proline, Bet: betaine, 2B: 1,2-butanediol.

HBA	HBD	Cosolvent	NAES/Cosolvent Ratio (%, *v/v*)
-	-	Water	0/100
-	-	Ethanol	0/100
ChCl	2B	-	100/0
ChCl	2B	Water	75/25
ChCl	2B	Water	50/50
ChCl	2B	Water	25/75
ChCl	2B	Ethanol	75/25
ChCl	2B	Ethanol	50/50
ChCl	2B	Ethanol	25/75
Pro	2B	-	100/0
Pro	2B	Water	75/25
Pro	2B	Water	50/50
Pro	2B	Water	25/75
Pro	2B	Ethanol	75/25
Pro	2B	Ethanol	50/50
Pro	2B	Ethanol	25/75
Bet	2B	-	100/0
Bet	2B	Water	75/25
Bet	2B	Water	50/50
Bet	2B	Water	25/75
Bet	2B	Ethanol	75/25
Bet	2B	Ethanol	50/50
Bet	2B	Ethanol	25/75

All mixtures were liquid and stable at room temperature.

## Data Availability

The data in this study are available in the article.

## Supplementary Materials

The following supporting information can be downloaded at https://www.mdpi.com/article/10.3390/molecules30183829/s1, Table S1: Density and dynamic viscosity values for all mixtures studied; Table S2: Values of conventional extraction (CE) for all mixtures studied; Table S3: Values of ultrasound-assisted extraction (USAE) for all mixtures studied; Table S4: Values of antioxidant activity of extracts and the percentage of degradation; Figure S1: HPLC chromatograms used for the identification and quantification of oleuropein; Figure S2: Calibration curve used for the quantification of oleuropein.
