# Supplementary material for: Ultrasound-Assisted Eutectic Solvent-Based Process Intensification for Sustainable Recovery of Oleuropein from Olive Leaves
Source: Molecules. 2025 Sep 21;30(18):3829. doi: 10.3390/molecules30183829 (PMC12472559; doi:10.3390/molecules30183829)
Supplement: Supplementary file 1 [file molecules-30-03829-s001.zip › molecules-3841842-supplementary.pdf]

Ultrasound-assisted eutectic solvent-based process intensification for sustainable recovery of oleuropein from olive leaves

Andrea Sánchez-Monedero, María González-Miquel, Emilio J. González*

Dpto. Ingeniería Química Industrial y del Medio Ambiente, ETSI Industriales, Universidad Politécnica de Madrid, C/ José Gutiérrez Abascal 2, Madrid, 28006, Spain.

*Corresponding author: maria.gonzalezmiquel@upm.es (María González Miquel)

Supplementary material

Table S1. Density and dynamic viscosity values for all mixtures studied.

Solvent	Composition (NAES-cosolvent, % (v/v)	Density, 25 °C (g/mL)	Density, 30 °C (g/mL)	Density, 40 °C (g/mL)	Dynamic viscosity, 25°C (mPa*s)	Dynamic viscosity, 30°C (mPa*s)	Dynamic viscosity, 40°C (mPa*s)
ChCl:2B-water	100-0	1.033	1.030	1.024	71.967	55.127	29.343
ChCl:2B-water	75-25	1.0371	1.0335	1.0275	12.817	9.980	6.860
ChCl:2B-water	50-50	1.0303	1.0272	1.0212	4.432	4.184	3.047
ChCl:2B-water	25-75	1.0142	1.012	1.0076	1.952	1.678	1.328
ChCl:2B-water	0-100	0.9956	0.9942	0.9922	1.091	0.945	0.713
ChCl:2B-ethanol	100-0	1.033	1.030	1.024	71.967	55.127	29.343
ChCl:2B-ethanol	75-25	0.9747	0.9718	0.9651	15.835	13.164	9.314
ChCl:2B-ethanol	50-50	0.911	0.9076	0.9005	5.434	4.780	4.034
ChCl:2B-ethanol	25-75	0.8456	0.8422	0.8337	2.509	2.334	1.870
ChCl:2B-ethanol	0-100	0.7843	0.7801	0.7717	1.189	1.094	0.887
Bet:2B-water	100-0	1.029	1.026	1.019	82.666	60.975	36.328

Bet:2B-water	75-25	1.0356	1.0318	1.0252	16.449	12.776	8.460
Bet:2B-water	50-50	1.0309	1.0242	1.0207	5.464	4.441	3.311
Bet:2B-water	25-75	1.0147	1.0126	1.0078	2.175	1.912	1.447
Bet:2B-water	0-100	0.9956	0.9942	0.9922	1.091	0.945	0.713
Bet:2B-ethanol	100-0	1.029	1.026	1.019	82.666	60.975	36.328
Bet:2B-ethanol	75-25	0.9689	0.9652	0.9576	20.145	15.818	10.604
Bet:2B-ethanol	50-50	0.913	0.9088	0.9013	6.222	5.346	3.890
Bet:2B-ethanol	25-75	0.8484	0.8443	0.8366	2.384	2.177	1.866
Bet:2B-ethanol	0-100	0.7843	0.7801	0.7717	1.189	1.094	0.887
Pro:2B-water	100-0	1.048	1.044	1.037	140.347	102.670	57.928
Pro:2B-water	75-25	1.0502	1.0465	1.0397	20.613	14.235	9.452
Pro:2B-water	50-50	1.0424	1.0389	1.0326	6.345	5.202	3.749
Pro:2B-water	25-75	1.022	1.0196	1.0152	2.300	1.972	1.505
Pro:2B-water	0-100	0.9956	0.9942	0.9922	1.091	0.945	0.713
Pro:2B-ethanol	100-0	1.048	1.044	1.037	140.347	102.670	57.928

Pro:2B-ethanol	75-25	0.9842	0.9804	0.9732	36.210	32.006	19.865
Pro:2B-ethanol	50-50	0.9211	0.917	0.9084	7.290	6.212	4.535
Pro:2B-ethanol	25-75	0.8539	0.8497	0.8415	2.509	2.231	1.779
Pro:2B-ethanol	0-100	0.7843	0.7801	0.7717	1.189	1.094	0.887

Table S2. Values of conventional extraction (CE) for all mixtures studied.

Solvent	Composition (NAES-cosolvent, % (v/v)	15 minutes, mean values ± SD (mg oleuropein / g ds)	30 minutes, mean values ± SD (mg oleuropein / g ds)	60 minutes, mean values ± SD (mg oleuropein / g ds)	100 minutes, mean values ± SD (mg oleuropein / g ds)
ChCl:2B-water	100-0	2.25 ± 0.08	17.50 ± 3.15	16.09 ± 2.90	10.13 ± 1.80
ChCl:2B-water	75-25	29.09 ± 5.24	33.60 ± 6.05	31.86 ± 5.74	30.29 ± 3.70
ChCl:2B-water	50-50	28.79 ± 0.51	31.48 ± 0.37	28.18 ± 5.07	24.40 ± 4.39
ChCl:2B-water	25-75	3.24 ± 0.58	7.12 ± 4.11	4.74 ± 0.85	2.75 ± 0.49
ChCl:2B-water	0-100	0.99 ± 0.18	0.87 ± 0.16	0.64 ± 0.12	0.48 ± 0.09
ChCl:2B-ethanol	100-0	2.25 ± 0.08	17.50 ± 3.15	16.09 ± 2.90	10.13 ± 1.82
ChCl:2B-ethanol	75-25	17.64 ± 3.17	21.38 ± 3.85	19.20 ± 3.46	13.79 ± 2.48
ChCl:2B-ethanol	50-50	25.82 ± 4.65	27.83 ± 5.01	25.75 ± 2.26	20.10 ± 3.62
ChCl:2B-ethanol	25-75	35.88 ± 6.46	38.13 ± 6.86	33.75 ± 6.07	27.10 ± 1.71
ChCl:2B-ethanol	0-100	30.08 ± 5.41	32.08 ± 5.78	26.66 ± 4.80	24.13 ± 3.96
Bet:2B-water	100-0	2.62 ± 0.11	23.30 ± 4.19	16.83 ± 2.64	13.36 ± 8.33
Bet:2B-water	75-25	31.01 ± 5.58	37.77 ± 2.00	34.92 ± 6.29	24.72 ± 1.95
Bet:2B-water	50-50	25.33 ± 4.56	35.38 ± 6.37	20.44 ± 3.68	13.76 ± 2.48
Bet:2B-water	25-75	2.44 ± 0.44	4.98 ± 0.90	4.46 ± 0.80	2.17 ± 0.39
Bet:2B-water	0-100	0.99 ± 0.18	0.87 ± 0.16	0.64 ± 0.12	0.48 ± 0.09

Bet:2B-ethanol	100-0	2.62 ± 0.11	23.30 ± 4.19	16.83 ± 2.64	13.36 ± 8.33
Bet:2B-ethanol	75-25	26.12 ± 4.70	29.72 ± 5.35	22.67 ± 4.08	21.93 ± 2.76
Bet:2B-ethanol	50-50	31.45 ± 5.66	39.57 ± 7.12	29.11 ± 1.32	26.96 ± 8.49
Bet:2B-ethanol	25-75	34.01 ± 6.12	40.03 ± 7.21	34.81 ± 6.27	30.53 ± 3.77
Bet:2B-ethanol	0-100	30.08 ± 5.41	32.08 ± 5.78	26.66 ± 4.80	24.13 ± 3.96
Pro:2B-water	100-0	2.22 ± 0.53	23.96 ± 4.31	22.23 ± 4.00	12.04 ± 2.17
Pro:2B-water	75-25	29.39 ± 5.29	39.83 ± 7.17	33.71 ± 6.07	31.49 ± 1.67
Pro:2B-water	50-50	20.41 ± 3.67	25.94 ± 4.67	24.13 ± 4.34	19.56 ± 3.52
Pro:2B-water	25-75	4.70 ± 0.85	17.38 ± 3.13	3.91 ± 0.70	2.67 ± 0.48
Pro:2B-water	0-100	0.99 ± 0.18	0.87 ± 0.16	0.64 ± 0.12	0.48 ± 0.09
Pro:2B-ethanol	100-0	2.22 ± 0.53	23.96 ± 4.31	22.23 ± 4.00	12.04 ± 2.17
Pro:2B-ethanol	75-25	24.81 ± 4.46	30.38 ± 5.47	23.30 ± 4.45	21.55 ± 3.88
Pro:2B-ethanol	50-50	28.37 ± 5.11	36.19 ± 6.51	25.46 ± 4.58	24.45 ± 4.40
Pro:2B-ethanol	25-75	28.59 ± 5.15	40.62 ± 7.31	33.89 ± 6.10	28.17 ± 1.48
Pro:2B-ethanol	0-100	30.08 ± 5.41	32.08 ± 5.78	26.66 ± 4.80	24.13 ± 3.96

Table S3. Values of ultrasound-assisted extraction (USAE) for all mixtures studied.

Solvent	Composition (NAES-cosolvent, % (v/v)	30 seconds, mean values ± SD (mg oleuropein / g ds)	60 seconds, mean values ± SD (mg oleuropein / g ds)	90 seconds, mean values ± SD (mg oleuropein / g ds)
ChCl:2B-water	100-0	8.33 ± 0.41	21.33 ± 4.27	13.11 ± 2.62
ChCl:2B-water	75-25	24.08 ± 3.95	30.26 ± 6.05	20.11 ± 5.31
ChCl:2B-water	50-50	22.32 ± 0.53	29.30 ± 5.86	18.97 ± 7.89
ChCl:2B-water	25-75	9.11 ± 1.82	12.14 ± 2.43	4.82 ± 0.96
ChCl:2B-water	0-100	1.22 ± 0.24	3.31 ± 0.66	1.16 ± 0.23
ChCl:2B-ethanol	100-0	8.33 ± 0.41	21.33 ± 4.27	13.11 ± 2.62
ChCl:2B-ethanol	75-25	18.05 ± 3.61	26.29 ± 5.26	15.22 ± 3.04
ChCl:2B-ethanol	50-50	24.90 ± 4.98	31.76 ± 10.46	17.86 ± 3.57
ChCl:2B-ethanol	25-75	30.90 ± 5.68	36.59 ± 7.32	23.94 ± 8.95

ChCl:2B-ethanol	0-100	24.45 ± 4.89	29.07 ± 5.81	17.10 ± 3.42
Bet:2B-water	100-0	7.09 ± 1.60	21.41 ± 2.69	8.05 ± 3.10
Bet:2B-water	75-25	20.84 ± 2.42	33.83 ± 3.84	12.52 ± 6.11
Bet:2B-water	50-50	18.54 ± 3.71	26.45 ± 6.91	9.20 ± 1.84
Bet:2B-water	25-75	9.90 ± 1.98	21.76 ± 4.35	1.29 ± 0.26
Bet:2B-water	0-100	1.22 ± 0.24	3.31 ± 0.66	1.16 ± 0.23
Bet:2B-ethanol	100-0	7.09 ± 1.60	21.41 ± 2.69	8.05 ± 1.61
Bet:2B-ethanol	75-25	17.13 ± 2.75	23.19 ± 4.64	16.19 ± 3.24
Bet:2B-ethanol	50-50	22.20 ± 0.95	25.82 ± 1.51	17.76 ± 3.55
Bet:2B-ethanol	25-75	24.66 ± 2.14	36.11 ± 8.92	19.22 ± 3.84
Bet:2B-ethanol	0-100	24.45 ± 4.89	29.07 ± 5.81	17.10 ± 3.42
Pro:2B-water	100-0	5.75 ± 2.14	11.59 ± 2.32	9.19 ± 1.84
Pro:2B-water	75-25	25.10 ± 1.11	33.32 ± 2.33	19.99 ± 4.00
Pro:2B-water	50-50	22.16 ± 9.39	30.31 ± 9.58	15.59 ± 3.12
Pro:2B-water	25-75	16.92 ± 3.38	27.71 ± 5.54	7.47 ± 1.49
Pro:2B-water	0-100	1.22 ± 0.24	3.31 ± 0.66	1.16 ± 0.23
Pro:2B-ethanol	100-0	5.75 ± 2.14	11.59 ± 2.32	9.19 ± 1.84
Pro:2B-ethanol	75-25	18.38 ± 3.28	31.98 ± 6.40	13.37 ± 2.67
Pro:2B-ethanol	50-50	23.99 ± 4.80	33.58 ± 6.72	17.32 ± 3.46
Pro:2B-ethanol	25-75	26.56 ± 2.71	37.00 ± 7.40	18.31 ± 3.66
Pro:2B-ethanol	0-100	24.45 ± 4.89	29.07 ± 5.81	17.10 ± 3.42

Table S4. Values of antioxidant activity of extracts and the percentage of degradation

Solvent	Measurement	Day 0	Day 3	Day 7	Day 14	Day 21	Day 31
Water	Antioxidant activity, mean values ± SD (%)	33.2 ± 4.6	22.2 ± 0.4	21.8 ± 0.8	17.6 ± 1.9	14.5 ± 1.7	14.5 ± 2.3
	Degradation, mean values ± SD (%)	0.00 ± 13.9	33.2 ± 1.1	34.2 ± 2.4	47.1 ± 5.7	56.4 ± 5.2	56.4 ± 7.0
Ethanol	Antioxidant activity, mean values ± SD (%)	56.0 ± 2.3	50.0 ± 5.3	49.8 ± 5.2	50.4 ± 7.5	46.2 ± 9.1	40.4 ± 7.7

	Degradation, mean values ± SD (%)	0.00 ± 4.1	10.7 ± 9.4	11.1 ± 9.2	10.1 ± 13.5	17.6 ± 16.2	27.9 ± 13.7
ChCl:2B-water, 75-25 % (v/v)	Antioxidant activity, mean values ± SD (%)	74.5 ± 7.4	72.0 ± 8.8	67.5 ± 6.5	68.8 ± 8.5	69.1 ± 7.7	67.8 ± 7.4
	Degradation, mean values ± SD (%)	0.00 ± 10.0	3.4 ± 11.8	9.4 ± 8.7	7.6 ± 11.4	7.3 ± 10.3	9.0 ± 9.9
ChCl:2B-ethanol, 25-75 % (v/v)	Antioxidant activity, mean values ± SD (%)	60.5 ± 0.4	57.0 ± 5.1	55.5 ± 5.8	57.7 ± 7.6	56.7 ± 9.9	52.3 ± 7.9
	Degradation, mean values ± SD (%)	0.00 ± 0.7	5.8 ± 8.4	8.3 ± 9.6	4.7 ± 12.6	6.3 ± 16.4	13.6 ± 13.0
Bet:2B-water, 75-25 % (v/v)	Antioxidant activity, mean values ± SD (%)	78.0 ± 3.8	75.0 ± 7.8	76.2 ± 6.4	74.6 ± 7.3	72.8 ± 8.0	63.0 ± 8.8
	Degradation, mean values ± SD (%)	0.00 ± 4.9	3.8 ± 10.0	2.3 ± 8.2	4.4 ± 9.4	6.6 ± 10.3	19.2 ± 11.3
Bet:2B-ethanol, 25-75 % (v/v)	Antioxidant activity, mean values ± SD (%)	57.7 ± 3.5	50.0 ± 7.3	51.4 ± 6.3	53.3 ± 6.6	52.7 ± 6.9	48.9 ± 7.2
	Degradation, mean values ± SD (%)	0.00 ± 6.0	13.3 ± 12.6	11.0 ± 11.0	7.7 ± 11.4	8.7 ± 12.0	15.3 ± 12.5
Pro:2B-water, 75-25 % (v/v)	Antioxidant activity, mean values ± SD (%)	90.0 ± 0.8	83.3 ± 0.2	79.9 ± 0.2	86.8 ± 0.0	87.0 ± 0.0	87.7 ± 0.0
	Degradation, mean values ± SD (%)	0.00 ± 0.8	7.1 ± 0.2	11.2 ± 0.02	3.6 ± 0.0	3.3 ± 0.0	2.6 ± 0.1
Pro:2B-ethanol, 25-75 % (v/v)	Antioxidant activity, mean values ± SD (%)	89.0 ± 2.7	81.8 ± 0.0	80.2 ± 0.0	86.1 ± 0.0	87.0 ± 0.3	87.4 ± 0.3
	Degradation, mean values ± SD (%)	0.00 ± 3.1	8.0 ± 0.0	9.9 ± 0.0	3.2 ± 0.0	2.2 ± 0.3	1.8 ± 0.3

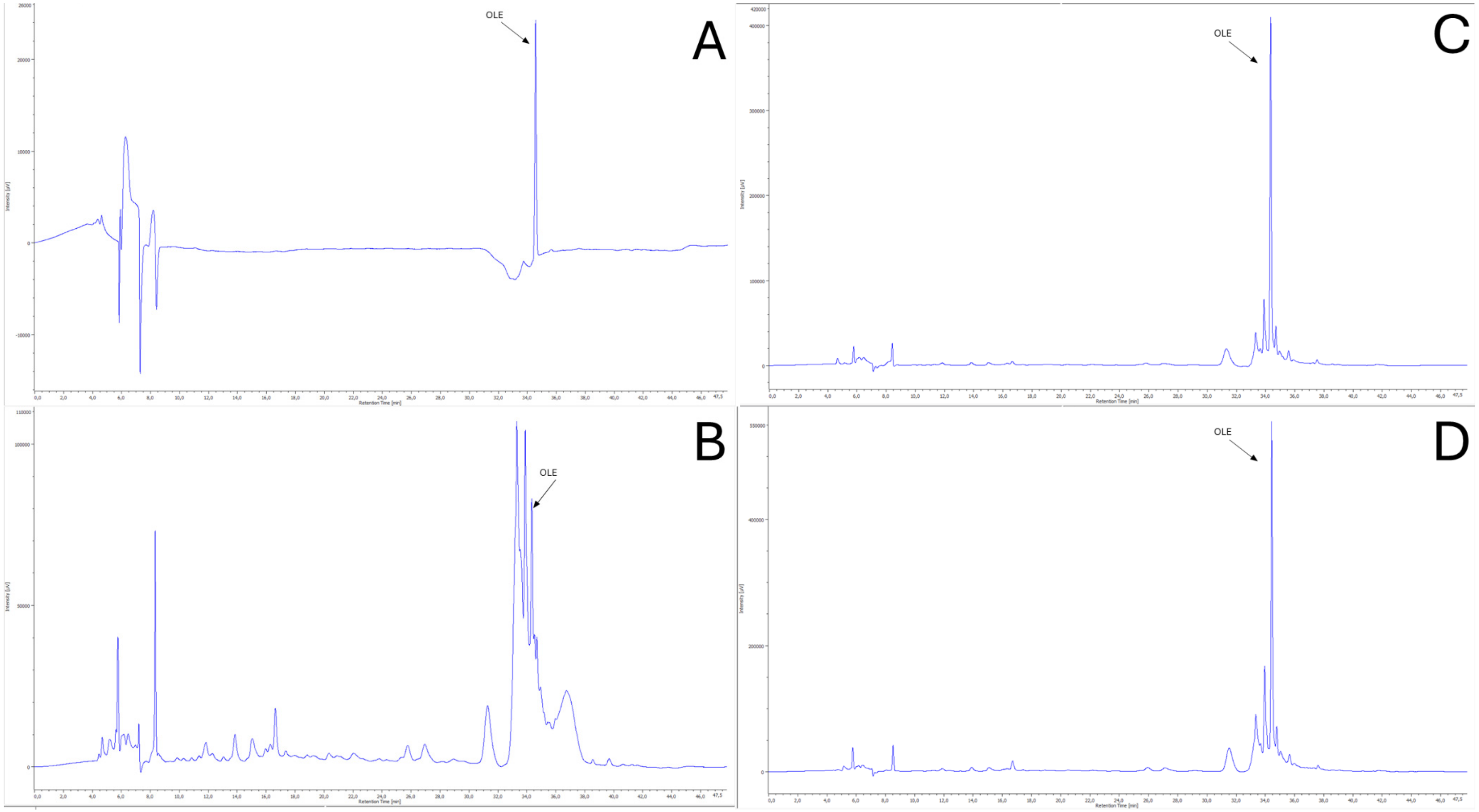

Figure S1. HPLC chromatograms used for the identification and quantification of oleuropein: (**A**) oleuropein standard at 20 ppm, (**B**) ultrasound-assisted extraction at 1 minute with water, (**C**) ultrasound-assisted extraction at 1 minute with ethanol, and (**D**) ultrasound-assisted extraction at 1 minute with the NAES/cosolvent mixture of Pro:2B/ethanol 25/75.

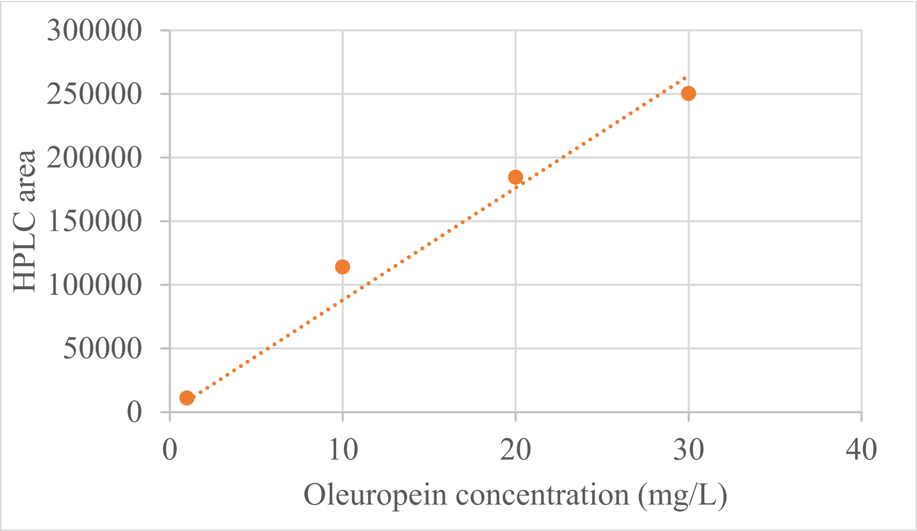

Figure S2. Calibration curve used for the quantification of oleuropein (R^2 = 0.9914)
